# Key Stakeholder Barriers and Facilitators to Implementing Remote Monitoring Technologies: Protocol for a Mixed Methods Analysis

**DOI:** 10.2196/38437

**Published:** 2022-07-21

**Authors:** Fahad Mujtaba Iqbal, Meera Joshi, Sadia Khan, Mike Wright, Hutan Ashrafian, Ara Darzi

**Affiliations:** 1 Division of Surgery Imperial College London London United Kingdom; 2 West Middlesex University Hospital London United Kingdom; 3 Chelsea and Westminster National Health Service Foundation Trust London United Kingdom

**Keywords:** implementation science, health plan implementation, mobile health, health care industry, stakeholder, barriers, remote monitoring, implementation, digitization, digital solutions

## Abstract

**Background:**

The implementation of novel digital solutions within the National Health Service has historically been challenging. Since the start of the COVID-19 pandemic, there has been a greater push for digitization and for operating remote monitoring solutions. However, the implementation and widespread adoption of this type of innovation have been poorly studied.

**Objective:**

We aim to investigate key stakeholder barriers and facilitators to implementing remote monitoring solutions to identify factors that could affect successful adoption.

**Methods:**

A mixed methods approach will be implemented. Semistructured interviews will be conducted with high-level stakeholders from industry and academia and health care providers who have played an instrumental role in, and have prior experience with, implementing digital solutions, alongside the use of an adapted version of the Technology Acceptance Model questionnaire.

**Results:**

Enrollment is currently underway, having started in February 2022. It is anticipated to end in July 2022, with data analysis scheduled to commence in August 2022.

**Conclusions:**

The results of our study may highlight key barriers and facilitators to implementing digital remote monitoring solutions, thereby allowing for improved widespread adoption within the National Health Service in the future.

**Trial Registration:**

ClinicalTrials.gov NCT05321004; https://clinicaltrials.gov/ct2/show/NCT05321004

## Introduction

Advancements in digital technologies, alongside increasing digitization in other industries and the global pandemic of COVID-19, have accelerated the adoption of novel health care pathways worldwide, with health care delivery transitioning beyond the traditional face-to-face model [1,2]. Telemedicine has gained long overdue exposure during a complicated crisis; since the onset of the pandemic, digital modalities have become critical in moderating exposure risk to health care staff, reducing community spread, and delivering quality health care remotely to exposed or infected individuals [3,4].

Remote monitoring solutions are being established internationally to allow individuals to continue living at home rather than in expensive hospital facilities, using noninvasive digital technologies (eg, wearable sensors) to collect health data and support health care provider assessment and clinical decision-making [5-9].

Wearable sensors, including patches, smart watches, clothing, and bands, can continuously register vital parameters (eg, heart rate, respiratory rate, blood pressure, oxygen saturation, and temperature) [10-14]. With the advances in technology miniaturization, sensors have become increasingly portable, unobtrusive, lightweight, and waterproof, offering an emerging solution to the continuous remote monitoring of vital signs. It is predicated that continuous monitoring will offer an opportunity for earlier clinical intervention through the earlier recognition of clinical deterioration, thereby improving patient care and patient outcomes, although it remains unclear whether the need for continuous monitoring for ambulatory patients is clinically meaningful [15].

Patient attitudes toward continuous remote monitoring in acute settings have been previously studied [16-18]. Although the work lacks external validity and has inherent selection bias, an insight into barriers and facilitators of continuous remote monitoring has been provided. More recently, a study reported favorable staff perceptions on the use of remote monitoring technologies in an acute surgical ward. Despite this however, the same cohort found no clinical benefit with varied engagement from health care staff, suggesting the need for the further exploration of implementation issues [19,20]. Within the United Kingdom, widespread digital transformations are facilitated by National Health Service (NHS) Digital—a nondepartmental public body created by statute that delivers large health informatics programs [21,22].

Digitally ready services, mature services, and data-enabled services are three ambitions listed by NHS England that form the basis of a digital framework for supporting digitization [23]. In addition, NHS England has supported virtual ward implementation, further indicating the future push toward digitization [24]. Therefore, there is a pressing need to understand key stakeholder perceptions to ensure the successful implementation of such services.

As such, our study aims to investigate key stakeholder perspectives, at the organizational level, on implementing remote monitoring solutions, given the pandemic, in the NHS to identify factors that could affect successful execution and adoption.

## Methods

### Study Design

A mixed methods approach will be implemented; semistructured interviews will be conducted with high-level stakeholders from industry, academia, and health care providers who have played an instrumental role in, and have prior experience with, implementing digital solutions. These individuals will be identified through their notable work with remote monitoring in health care (eg, authors of impactful research in the literature, major digital technology companies, and experts recommended by peers). The purpose of the interviews will be to highlight barriers and facilitators to the implementation process. This will allow for a road map to be created for the future deployment of digital solutions. In conjunction, questionnaires will be undertaken to determine the perceived technological acceptance of new remote monitoring systems.

### Ethics Approval

All recruited participants will provide informed consent. Ethical approval for this study was granted by the Imperial College London's Science, Engineering and Technology Research Ethics Committee (reference number: 20IC6331). The trial will be performed in accordance with good clinical practice guidelines and the Declaration of Helsinki. Patient data will be anonymized to ensure privacy. The storage and handling of personal data will comply with the General Data Protection Regulation.

### Questionnaires

An adapted version of the Technology Acceptance Model (TAM) questionnaire will be used (Multimedia Appendix 1). This version was previously validated, and it demonstrated acceptably high Cronbach α values [25]. The proposed theoretical framework (information technology acceptance) was adapted from Chau and Hu [26], comprising individual context, technological context, and organizational context. Gagnon et al [25] adapted this further with the inclusion of the theories of interpersonal behavior and reasoned action, building on the TAM proposed by Davis [25-29]. As such, individual context consists of compatibility (factors that affect the acceptance of a new technology) and attitude (a perception of an individual toward adopting a technology), and technological context consists of the perceived usefulness and perceived ease of use of technologies, alongside habits of individuals. Lastly, organization context consists of facilitators and subjective norms; the latter can be described as *social* (an individual’s perception toward a behavior) or *descriptive* (the behaviors of others).

### Semistructured Interviews

All participants will be invited to take part in semistructured interviews conducted by the lead researcher (FI). A structured topic guide was created (Multimedia Appendix 1) by following a literature review that drew heavily from a model proposed by Simblett et al [30]. The guide highlights the following five key areas for determining the likelihood of engagement with remote monitoring technology by stakeholders: health status, usability, convenience/accessibility, perceived utility, and motivation. Data collection will be an iterative process; emerging recurring concepts will be incorporated into the interview guide for further exploration with remaining participants. Interviews will then be recorded, anonymized, and transcribed verbatim before being entered into NVivo 12 (QSR International) for analysis.

### Statistical Analysis

Frequency distributions will be used to show responses to the TAM questionnaire. Responses will be recorded by using a 7-point Likert scale (“strongly disagree” to “strongly agree”). Analyses will be performed by using R Studio (RStudio, PBC).

Transcribed interviews will be analyzed by using a broadly deductive approach [31], with the adapted topic guide described by Simblett et al [30], which will form the basis for the initial predefined coding framework. This will be undertaken by two independent researchers to determine barriers and facilitators [31]. An iterative process of coding and data indexing will occur, ensuring that key aspects are not missed from the predefined coding framework. Subsequent emerging themes will be summarized thereafter. The results will be discussed until consensus is reached. Interviews will be analyzed until data saturation is achieved.

## Results

Enrollment is currently underway, having started in February 2022. It is anticipated to end in July 2022, with data analysis scheduled to occur in August 2022.

## Discussion

Our study has the potential to identify barriers and facilitators of implementing remote monitoring solutions within an NHS trust. It will lay a road map based on the collated experiences of key stakeholders for the future deployment of remote monitoring solutions, thereby improving widespread adoption.

Indeed, a recent study highlighted the effectiveness of such solutions in patients with COVID-19, although the study noted the limited number of high-quality trial designs, which were heterogenous in nature [32]. The pandemic has resulted in heightened interest in public health research with models for implementing pulse oximetry. Given this digital acceleration, further research into implementation strategies are of growing importance [33,34].

Our study will be limited to the implementation of remote monitoring solutions, and the findings may not be generalizable to other digital solutions nor to other health care settings. Moreover, nonprobabilistic sampling may result in selection bias. Despite this, our use of semistructured interviews to capture stakeholder perceptions may yield pertinent considerations for the pragmatic implementation of remote monitoring, and the broad heterogenous sample of key stakeholders we hope to include may identify issues that are generalizable to the implementation of other technologies—an area of paucity within the current literature and an area of growing importance, given the favorable patient attitudes toward continuous remote monitoring [16-18]. Lastly, the viewpoints of end users will not be examined in this study, as this has been done elsewhere [19,35]. As such, a top-down view has been provided herein.

In conclusion, the results of our study could offer insight into highlighting key barriers and facilitators to implementing digital remote monitoring solutions, thereby allowing for improved widespread adoption within the NHS in the future.

## Appendix

Multimedia Appendix 1 Modified Technology Acceptance Model questionnaire and semistructured interview questions.

## Abbreviations

NHS: National Health Service
NIHR: National Institute for Health and Care Research
TAM: Technology Acceptance Model

## Data Availability

The data sets used and/or analyzed during the study will be available from the corresponding author on reasonable request.
