# Supplementary material for: Key Stakeholder Barriers and Facilitators to Implementing Remote Monitoring Technologies: Protocol for a Mixed Methods Analysis
Source: JMIR Res Protoc. 2022 Jul 21;11(7):e38437. doi: 10.2196/38437 (PMC9353674; doi:10.2196/38437)
Supplement: Multimedia Appendix 1 [file resprot_v11i7e38437_app1.pdf]

Evaluation of perceived acceptance of virtual wards

Please spend no more than 30 seconds on each question, the aim of this work is to better understand the perspectives at an organisational level for the readiness, preparedness, and acceptance of using remote monitoring and virtual wards.

Definitions:
Virtual ward – an online portal which is monitored by healthcare professionals with patients admitted ‘virtually’
Remote monitoring – the use of sensors to measure and record vital sign monitoring continuously remotely

---

* Required

1. Age *

____

2. Sex *

Mark only one oval.

- ( ) Male
- ( ) Female

3. Job role *

Mark only one oval.

- ( ) Clinical
- ( ) Non-clinical

4. Most people will be comfortable with information and communication technologies *

Mark only one oval.

	1	2	3	4	5	6	7	
Strongly disagree	◯	◯	◯	◯	◯	◯	◯	Strongly agree

5. The use of virtual wards could help clinicians monitor patients more rapidly *

Mark only one oval.

	1	2	3	4	5	6	7	
Strongly disagree	◯	◯	◯	◯	◯	◯	◯	Strongly agree

6. It is a good idea to use continuous remote monitoring in the community *

Mark only one oval.

	1	2	3	4	5	6	7	
Strongly disagree	◯	◯	◯	◯	◯	◯	◯	Strongly agree

7. Virtual wards may imply major changes to clinical practice beyond the pandemic *

Mark only one oval.

	1	2	3	4	5	6	7	
Strongly disagree	◯	◯	◯	◯	◯	◯	◯	Strongly agree

8. It would be easy to perform the tasks necessary for continuous remote monitoring of patients using remote monitoring solutions *

Mark only one oval.

	1	2	3	4	5	6	7	
Strongly disagree	◯	◯	◯	◯	◯	◯	◯	Strongly agree

9. Most patients will welcome the virtual ward model *

Mark only one oval.

	1	2	3	4	5	6	7	
Strongly disagree	◯	◯	◯	◯	◯	◯	◯	Strongly agree

10. The necessary infrastructure to support virtual wards can be easily put in place *

Mark only one oval.

	1	2	3	4	5	6	7	
Strongly disagree	◯	◯	◯	◯	◯	◯	◯	Strongly agree

11. Virtual wards will improve time efficiency of healthcare professionals monitoring patients *

Mark only one oval.

	1	2	3	4	5	6	7	
Strongly disagree	◯	◯	◯	◯	◯	◯	◯	Strongly agree

12. Clinical deterioration would be easy to detect and act on *

Mark only one oval.

	1	2	3	4	5	6	7	
Strongly disagree								Strongly agree

13. The intention to use remote monitoring when necessary to provide healthcare to patients is present *

Mark only one oval.

	1	2	3	4	5	6	7	
Strongly disagree								Strongly agree

14. Remote monitoring can facilitate the care of patients *

Mark only one oval.

	1	2	3	4	5	6	7	
Strongly disagree								Strongly agree

15. Remote monitoring may promote good clinical practice *

Mark only one oval.

	1	2	3	4	5	6	7	
Strongly disagree								Strongly agree

16. Early versions of remote monitoring (e.g. virtual clinics of telephone clinics) have been useful *

Mark only one oval.

	1	2	3	4	5	6	7	
Strongly disagree	⬭	⬭	⬭	⬭	⬭	⬭	⬭	Strongly agree

17. The use of continuous remote monitoring is beneficial for the care of patients *

Mark only one oval.

	1	2	3	4	5	6	7	
Strongly disagree	⬭	⬭	⬭	⬭	⬭	⬭	⬭	Strongly agree

18. It will be easy to acquire skills to use the remote monitoring system *

Mark only one oval.

	1	2	3	4	5	6	7	
Strongly disagree	⬭	⬭	⬭	⬭	⬭	⬭	⬭	Strongly agree

19. Allied healthcare professionals (e.g. nurses, physiotherapists) would welcome virtual wards *

Mark only one oval.

	1	2	3	4	5	6	7	
Strongly disagree	⬭	⬭	⬭	⬭	⬭	⬭	⬭	Strongly agree

20. Virtual wards will have a positive impact *

Mark only one oval.

	1	2	3	4	5	6	7	
Strongly disagree	◯	◯	◯	◯	◯	◯	◯	Strongly agree

21. Virtual wards would be well perceived with appropriate technical assistance *

Mark only one oval.

	1	2	3	4	5	6	7	
Strongly disagree	◯	◯	◯	◯	◯	◯	◯	Strongly agree

Semi-structured interview questions

What is your current role?
How long have you been in this role?
What do you understand by remote monitoring?
What do you understand by virtual ward?
Would you use remote monitoring or virtual ward interchangeably?
What are you attitudes towards remote monitoring?
Is it a distraction?
Is it another initiative that you feel will pass in due course
Do you have any experience where this has been used?
If so, where was it used?
What are the barriers of implementing remote monitoring or virtual wards?
What are the facilitators of implementing remote monitoring or virtual wards?
What are the major factors that lead to adoption and usage?
What human factors?
What technology factors?
What organisational factors?
What implementation strategies are required for successful adoption of digital technologies?
What cost issues do you anticipate?
